# Review of "Thrombosis in Clinical Practice"

**DOI:** 10.1186/1477-9560-3-7

**Published:** 2005-07-15

**Authors:** Raul Altman

As mentioned by the editors, the expectant readers of Thrombosis in Clinical Practice are physicians, general practitioners, nurse practitioners, and healthcare workers. With this in mind I browsed Thrombosis in Clinical Practice.

Thrombosis is a fast moving area and is not easy to give updated information of its pathophysiology and treatment in about 300 pages as it was successfully done in this book.

This multiauthored text combines basic concepts of mechanisms of thrombosis formation as well as therapeutic approaches.

It provides short overviews on risk factors, therapeutics and a systematic discussion of arterial and venous thrombosis management with final recommendations according to the authors experience. It successfully delivers concise information on all aspects of a given type of thrombosis for physicians and nurses who care for patients with thrombosis related diseases.

The first five chapters review the mechanism of thrombus formation (although importance of inflammation was omitted in Chapter 1), the role of the hemostatic system in arterial and venous thrombosis, characteristics of what the authors call "commonly used anticoagulant and antiplatelet drugs" (which include aspirin, heparin, and warfarin starting with an interesting historical perspective of each of them).

Without doubt the risk of bleeding is the most critical aspect of patients under antithrombotic treatment; "why it happens and what to do" are questions answered in Chapter 4.

Factors which determine thrombophilia are discussed in Chapter 5. Practical recommendations for screening patients with potential or already developed thrombotic process are noteworthy.

The following Chapters 6 to 12 deal with treatment of arterial and venous thrombosis and include management of atrial fibrillation where the known, well established therapies are discussed. In cardiac valves, Chapter 7, a comprehensive indication for the used of oral anticoagulant with or without aspirin and the management of pregnant patients was explained. Since some not so recent data have been included in the beginning of this chapter authors missed to mention that in patients with cardiac valves prostheses, the first combined therapy (oral anticoagulant plus dipyridamole) for thrombotic preventing treatment was published by Sullivan et al. (N Engl J Med. 1971;284:1391-4), and belongs to our group the first report on the use of aspirin plus oral anticoagulant (Altman et al. J.Thorac.Cardiov. Surg. 72: 127,1976).

Chapters 8 to 11 review the benefit and risk of antithrombotic therapy in coronary artery disease, thrombosis prevention after coronary interventions and, an updated discussion on risk factors, pathophysiology, diagnosis and antithrombotic therapy in peripheral arterial disease and ischemic stroke.

Chapter 12 deals with an important and still controversial topic: "Management of venous thromboembolism during pregnancy". The authors discuss the conflictive positions in the diagnostic as well as in the treatment of deep venous thrombosis in pregnant women. The antithrombotic treatment in women with recurrent miscarriage and thrombophilia which is conflictive and still unresolved is not discussed in this chapter.

Chapter 13 was dedicated to thrombophilia. I wonder why it was included at the end of the book and not among the first 5 Chapters. I also wonder why Ian Jennings omits to discuss whether the decrease of fibrinolysis is (or not) a potential thrombotic risk factor.

Importantly, a chapter on thrombosis in children was incorporated in this book and finally, a chapter on new drugs for anticoagulant therapy, which could be called "the after coumadin/heparin era", was also included with updated information on the pentasacharides fondaparinux and idraparinux, on drugs affecting factor VII activity, direct Factor Xa and direct thrombin inhibitors. Therapeutic characteristics of antiplatelet drugs is provide in this Chapter. This part of the article shows the feeble beneficial effect of clopidogrel plus aspirin, in reducing the combining risk of ischemic stroke, MI or vascular death compared with aspirin in several artery diseases and likely to be beneficial in patients underwent percutaneous coronary intervention. The antithrombotic capacity of glicoprotein IIb-IIIa (integrin α2b/β3) inhibitors, the potential value of von Willebrand factor antibody AJW 200 and thrombolytic therapy are also discussed.

This textbook is particularly interesting for general practitioners, medical students and trainees in haematology. It will be useful for physicians in several disciplines.

In short: It fulfils its stated purpose.

